# Supplementary material for: Template-in-template assembly nanostructured microspheres for high performance chromatography
Source: Nat Commun. 2026 Feb 4;17:430. doi: 10.1038/s41467-026-68362-y (PMC12873412; doi:10.1038/s41467-026-68362-y)
Supplement: Supplementary file 1 — Supplementary Information [file 41467_2026_68362_MOESM1_ESM.pdf]

Supplementary Information

Template-in-Template Assembly Nanostructured Microspheres for High Performance Chromatography

Author Information

Juxing Zeng[1, 2, †], Hanchen Cao[1, 2, †], Kaiyue Sun[1, 2, †], Zhuoheng Zhou[3, †], Lin Lv[1, 2], Jikai Chen[1, 2], Xiangyu Huang[1, 2], Xiaofei Wang[4], Bo Zhang[1, 2, *]

Affiliations

1. Department of Chemistry and the MOE Key Laboratory of Spectrochemical Analysis & Instrumentation, College of Chemistry and Chemical Engineering, Xiamen University, Xiamen 361005, China.

2. State Key Laboratory of Vaccines for Infectious Diseases, Xiang An Biomedicine Laboratory, Xiamen University, Xiamen 361005, China.

3. Bioanalytical Services, Department of Toxicology, WuXi AppTec (Suzhou) Co., Ltd, Suzhou 215000, China.

4. ColumnScientific Inc., Xiamen 361102, China.

†. These authors contributed equally to this work.

Contributions

J.Z., B.Z., and Z.Z. conceived the project and designed the experiments. J.Z., B.Z., H.C., and K.S. co-wrote the manuscript. J.Z., J.C., and X.H. carried out the synthesis and characterization of the materials. H.C., L.L., and X.W. involved in the application data collection. Z.Z., H.C., and K.S. assisted J.Z. for the data collection and analysis. All authors contributed to the discussion and manuscript preparation.

Corresponding author

*Bo Zhang. Phone/Fax: 0086-592-2188691. Email: bozhang@xmu.edu.cn.

Materials and Reagents. Thiourea, acetonitrile, ethanol, dichloromethane, hydrochloric acid (37.0-40.0%), methanol, toluene (dried over molecular sieves prior to use), *n*-hexane, acetylacetone, and potassium bromide were all analytical grade and purchased from Sinopharm Chemical Reagent Co., Ltd. Tetraethyl orthosilicate (TEOS), 1,2-bis(triethoxysilyl)ethane (BTEE), 1,4-bis(triethoxysilyl)benzene, titanium (IV) butoxide, zirconium (IV) butoxide (80 wt% in *n*-butanol), dimethyloctadecylchlorosilane (>97%), ethylbenzene (analytical standard), propylbenzene (analytical standard), butylbenzene (analytical standard), benzene (analytical standard), benzene-d6 (≥99.5 atom% D), 1,4-dibromobenzene (≥99%), PAH-Mix9 (US EPA 16, 200 μg/mL each component in acetonitrile), and xylene (≥97%, mixture of isomers) were purchased from Shanghai Aladdin Biochemical Technology Co., Ltd. 1,4-dibromobenzene-d4 was purchased from Shanghai Yien Chemical Technology Co., Ltd. Acetonitrile (HPLC grade), polyethylene glycol hexadecyl ether Brij® C10 (Brij 56, Mn~683), pluronic® P123 (P123, Mn~5800), and plurnoic®F127 (F127, Mn~12600) were purchased from Sigma-Aldrich (Shanghai) Trading Co., Ltd. Electronic fluorinated fluids FC-40 and HFE-7500 were procured from 3M Company (Minnesota, USA). The diblock copolymer PEO_{125}-*b*-$PMMA_{249}$ was synthesized via atom transfer radical polymerization (ATRP) according to a previously reported method[1]. The triblock copolymer PFPE-*b*-PEG-*b*-PFPE (PEG-polyethylene glycol, Mw~600 Da, PFPE-perfluoropolyether, Mw~7500 Da) was synthesized according to a previously reported method[2,3]. Ultrapure water (18.2 MΩ) was prepared in a Milli-Q system (Bedford, USA). All chemicals were used as received unless otherwise specified.

Apparatus. Harvard syringe pumps 11 Pico Plus Elite (Harvard Apparatus, USA) were used to drive dispersed and continuous phases in droplet microfluidics. The microfluidic chip was fabricated according to a previously reported method[4] and fixed on an inverted microscope Nikon L150 (Nikon, Japan), and a high-speed camera Phantom Miro C110 from Vision Research (AMETEK, USA) was equipped on the microscope to monitor the droplet generation. Hitachi S-4800 scanning electron microscope (Hitachi, Japan) was used to characterize the morphology of microspheres. JEM 1400 transmission electron microscopy (JEOL, Japan) was used to characterize the pore structure of microspheres. Micromeritics ASAP 2460 analyzer (Micromeritics, USA) was used to characterize the porosity. SAXSess mc^2 Small & Wide Angle X-Ray Scattering System (Anton Paar, Austria) was used to characterize the pore structure. Nicolet iS50 FTIR spectrometer (Thermofisher, USA) was used to characterize functional groups in organosilica. FlashSmart Elemental Analyzer (Thermal Fisher, USA) was used to characterize the carbon content in C18 bonded stationary phases. An Elite P230 high-pressure pump (Elite Analytical Instruments, China) was used for high-pressure slurry packing of capillary columns. The chromatographic performance was evaluated on Ultimate 3000 RSLCnano system (Thermo Scientific, Netherlands), equipped with a 4 nL Valco nanovolume injector (VICI, Switzerland) and a variable wavelength UV−vis detector with a 3 nL flow cell.

Characterization and Measurements. Droplet generation and evaporation characterization were performed by placing the microfluidic chip on an inverted microscope platform equipped with a high-

speed camera for real-time observation and recording of droplet formation, while the evaporation process was performed by monitoring collected droplets in a Petri dish under the same microscope. To characterize microspheres' morphology, the microspheres were dispersed in methanol, deposited onto silicon substrate, sputter-coated with platinum, and conducted on scanning electron microscope operated at 5 kV/10 μA, and the particle size distribution was statistically analyzed from 100 individual microspheres per sample using the open-source software Fiji. Transmission electron microscopy (TEM) pore structure analysis was performed by grinding microspheres, dispersing them in methanol, depositing 10 μL suspensions onto copper grids, drying, and observing with a JEM1400 TEM at 100 kV. The pore structure measurements were taken on SAXSess mc^2 Small & Wide-Angle X-Ray Scattering System using Cu Kα radiation (40 kV, 50 mA, 10 min acquisition), the *d*-spacing values were calculated by formula $d=2\pi/q$. The nitrogen sorption experiments were conducted at 77 K using Micromeritics ASAP 2460 analyzer. Before analyzing, the sample were degassed at 200°C for at least 6 h. The specific surface area was calculated via the BET method, pore size distribution and pore volume derived from adsorption branches using the BJH model, and pore volume evaluated from the adsorbed amount at a relative pressure P/P_0 of 0.995. IR characterization of organosilica functionalization was conducted on a Nicolet iS50 spectrometer. The carbon content of C18 modified stationary phases was characterized by FlashSmart Elemental Analyzer using CHNS-mode.

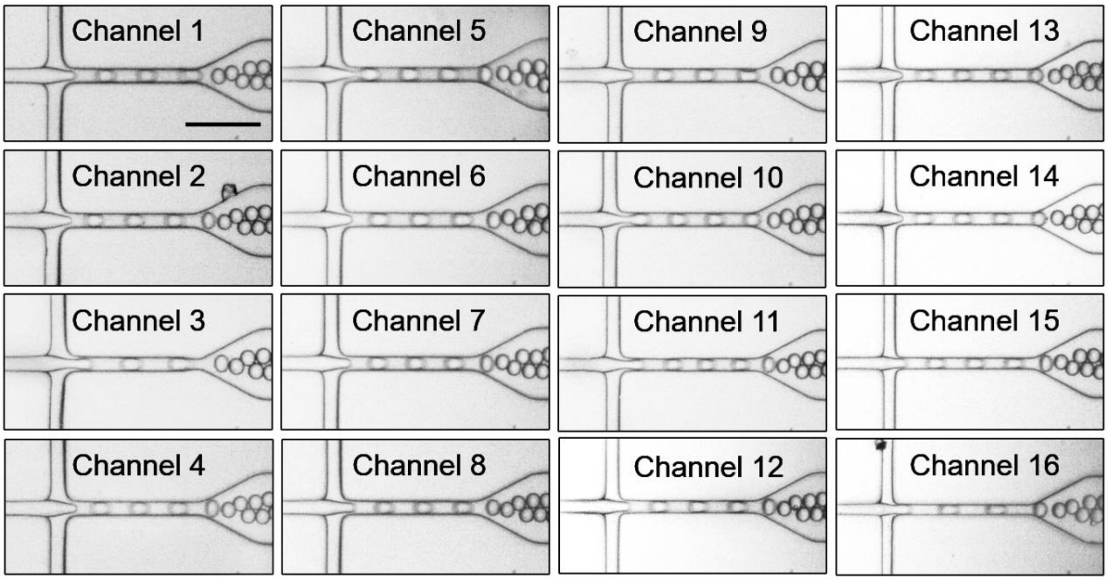

Supplementary Figure 1. Representative droplet generation in 120-channel array super-throughput chip.

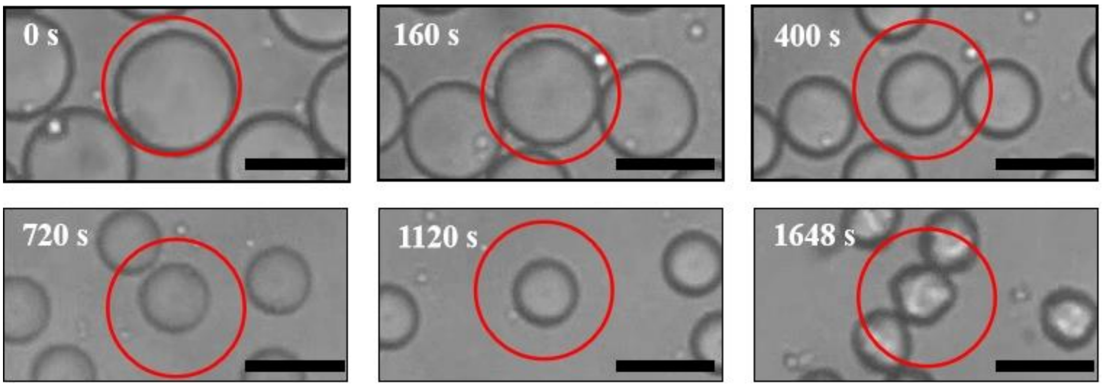

Supplementary Figure 2. Solvent evaporation of droplets in petri dish. The scale bar is 90 μm.

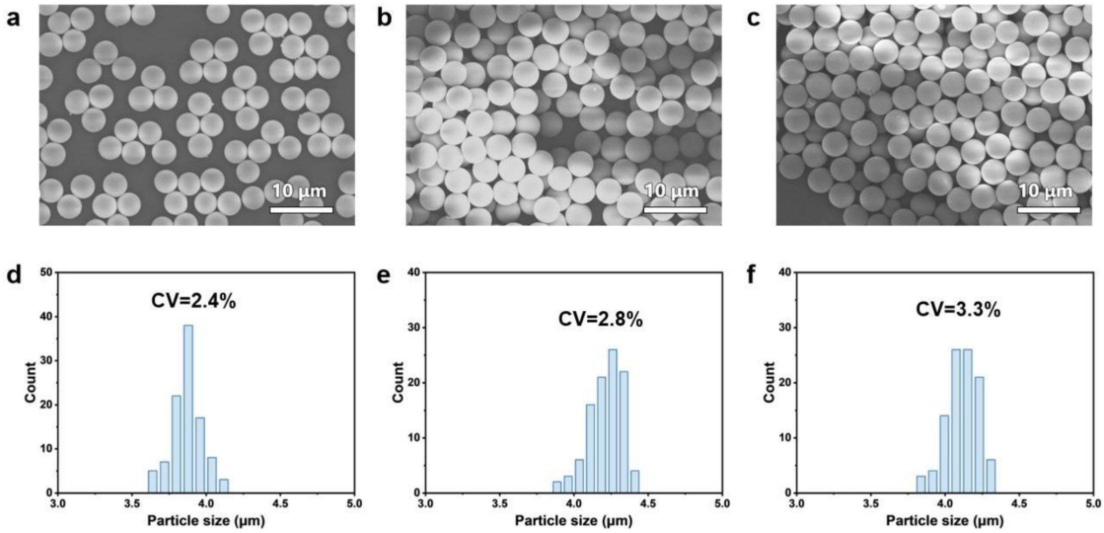

Supplementary Figure 3. Characterization of monodisperse microspheres with ordered mesopore structure. SEM morphology characterization and particle size distribution of ordered mesoporous silica microspheres **(a, d)** before hydrothermal treatment, **(b, e)** after hydrothermal treatment, and **(c, f)** after calcination.

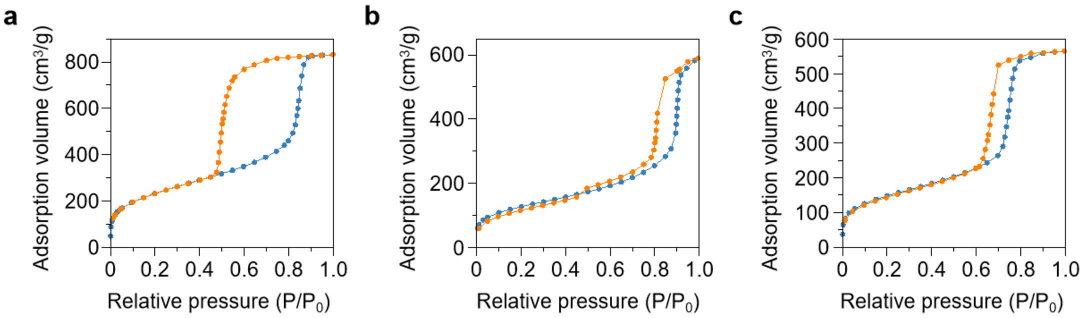

Supplementary Figure 4. Nitrogen adsorption and desorption isotherms of mesoporous silica microspheres with different ordered configurations templated by different SDAs. a Body centered cubic configuration templated by F127. **b** Face centered cubic configuration templated by PEO_{125}-*b*-$PMMA_{249}$. **c** Cubic double gyroid configuration templated by P123.

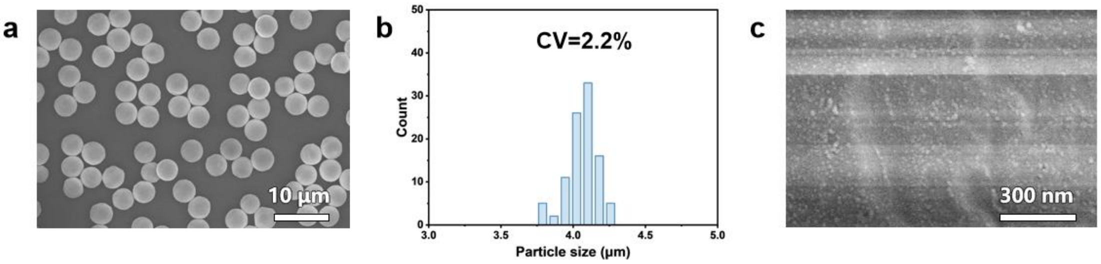

Supplementary Figure 5. Characterization of microspheres with cubic double gyroidal structure. **a, c** SEM morphology characterization with different magnifications and **(b)** particle size distribution of the cubic double gyroidal mesoporous silica microspheres templated by P123.

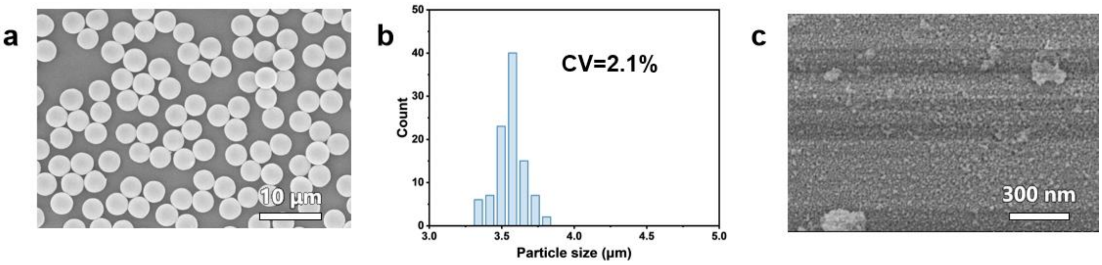

Supplementary Figure 6. Characterization of microspheres with body centered cubic structure. a, c SEM morphology characterization with different magnifications and **(b)** particle size distribution of the body centered cubic mesoporous silica microspheres templated by F127.

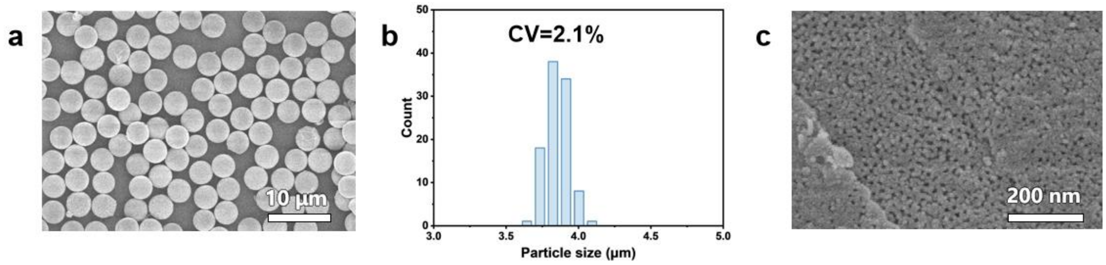

Supplementary Figure 7. Characterization of microspheres with face centered cubic structure. a, c SEM morphology characterization with different magnifications and **(b)** particle size distribution of the face centered cubic mesoporous silica microspheres templated by PEO_{125}-*b*-$PMMA_{249}$.

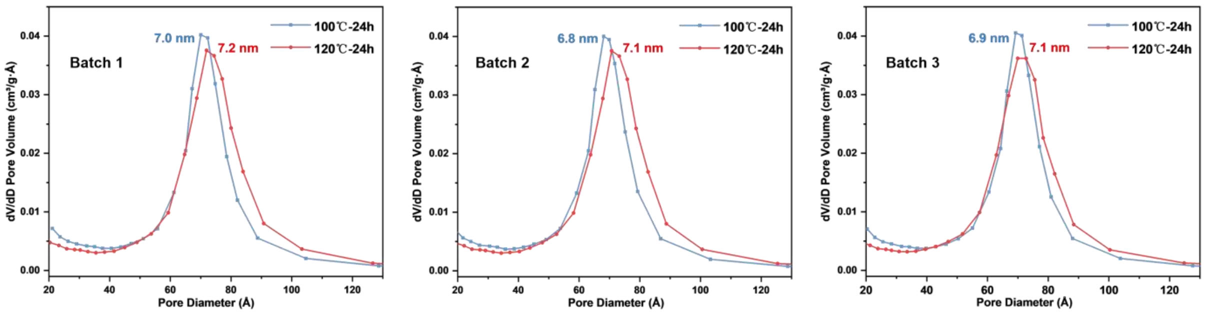

Supplementary Figure 8. Reproducibility of pore size tuning via hydrothermal treatment. Droplets from three independent batches were collected over three days, and each batch was divided into two groups subjected to different hydrothermal treatments.

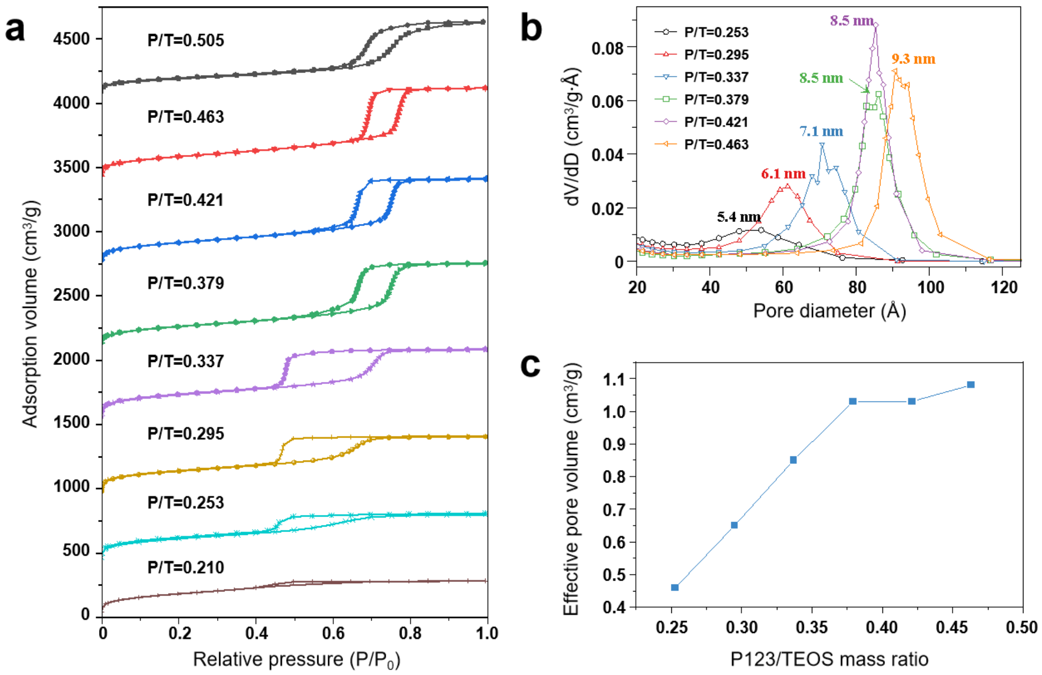

Supplementary Figure 9. Porosity characterization of ordered mesoporous silica microspheres templated by different P123 amounts (P/T = mass ratio of P123 to TEOS). a Nitrogen adsorption and desorption isotherms. **b** BJH adsorption pore size distribution. **c** Effective pore volume of ordered mesoporous silica microspheres templated by different P123 amounts.

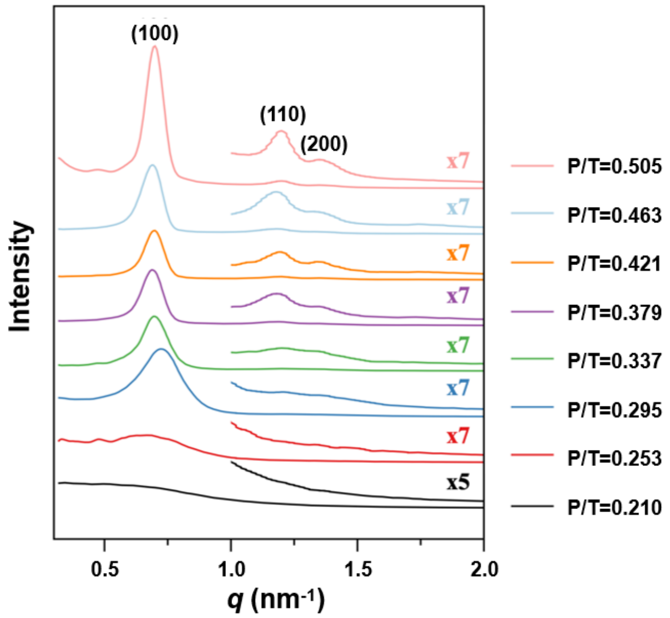

Supplementary Figure 10. SAXS patterns of ordered mesoporous silica microspheres templated with different P123 amounts (P/T = mass ratio of P123 to TEOS).

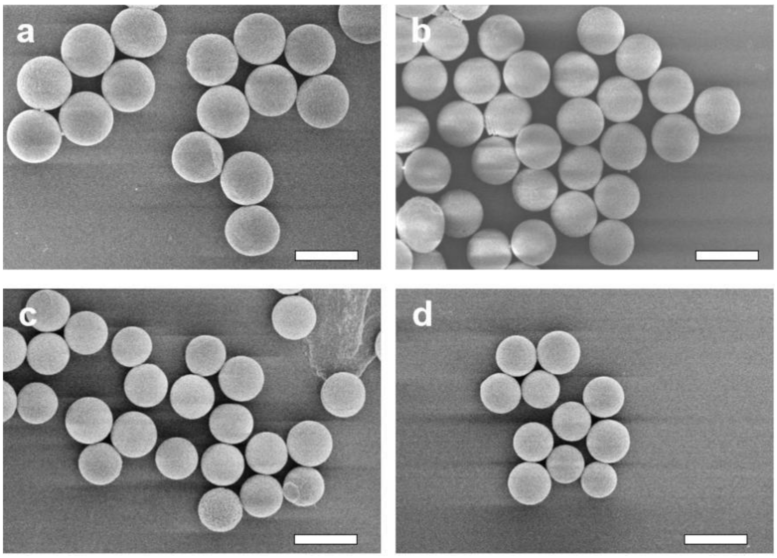

Supplementary Figure 11. **Effect of PEO_{125}-*b*-$PMMA_{249}$ amounts on mesoporous silica microspheres' morphology.** SEM images of mesoporous silica microspheres templated with **(a)** 10 wt%, **(b)** 20 wt%, **(c)** 30 wt%, and **(d)** 40 wt% PEO-b-PMMA (mass ratio: PEO_{125}-*b*-$PMMA_{249}$/TEOS). The scale bar is 10 μm.

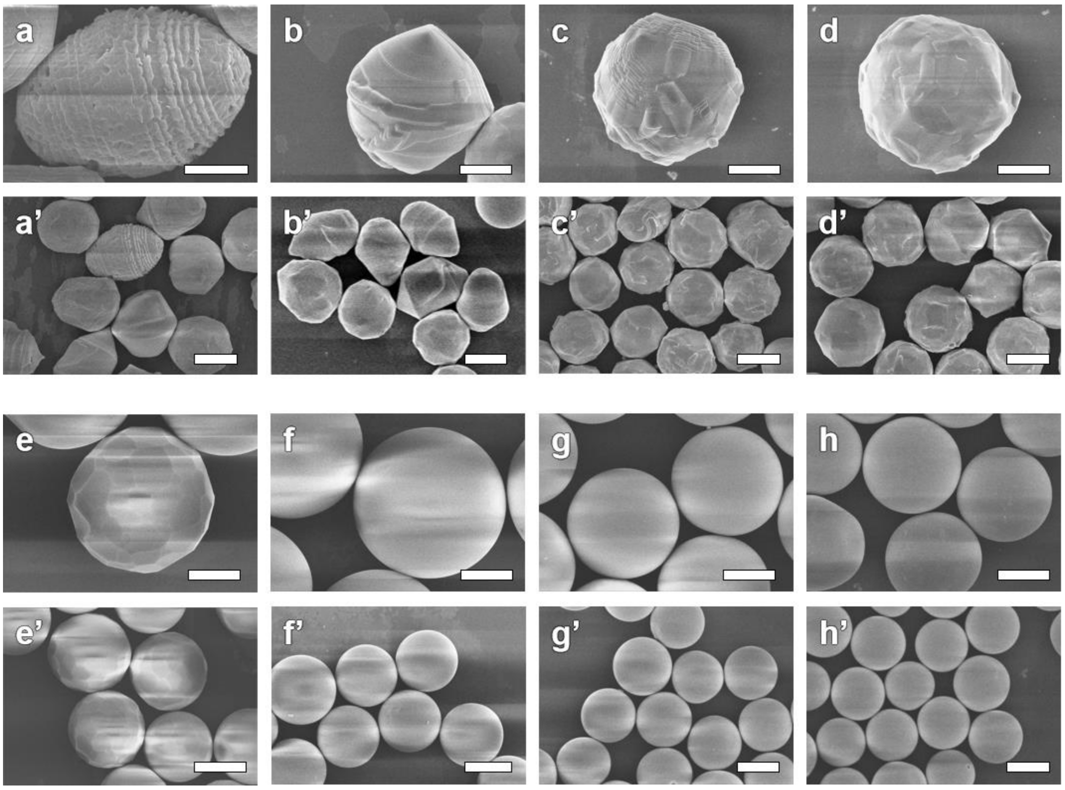

Supplementary Figure 12. Effect of P123 amounts on mesoporous silica microspheres’ morphology. SEM images of mesoporous silica microspheres templated with **(a)** 50 wt%, **(b)** 46 wt%, **(c)** 42 wt%, **(d)** 38 wt%, **(e)** 34 wt%, **(f)** 30 wt%, **(g)** 25 wt%, **(h)** 21 wt% P123 (mass ratio: P123/TEOS). With the increasing of P123 content, the morphology of mesoporous silica microspheres transformed from smooth sphere to sphere-like polyhedron, spindle, and to irregular shape. The scale bar is **(a - h)** 5 μm and **(a’ - h’)** 10 μm, respectively.

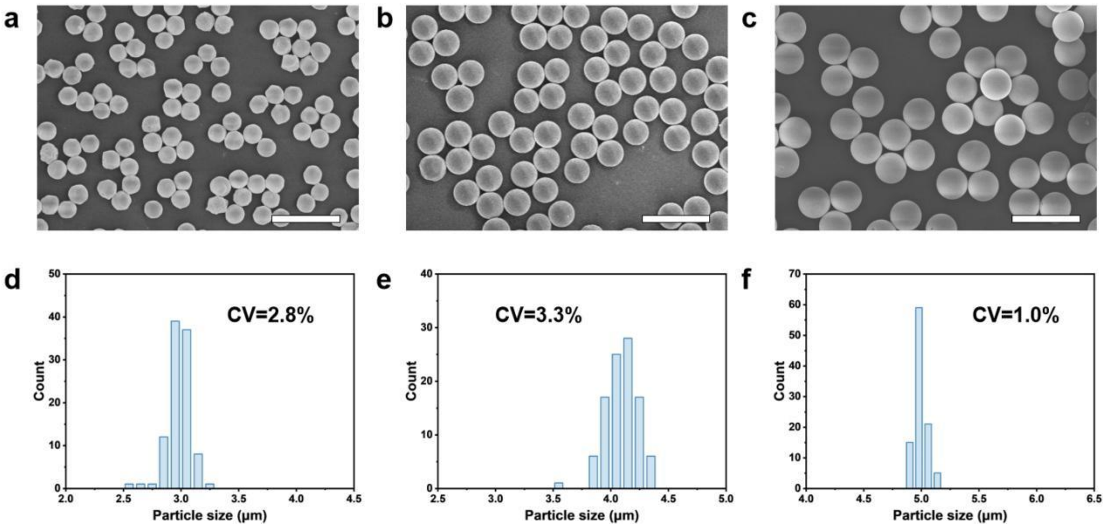

Supplementary Figure 13. Characterization of microspheres with different particle sizes. SEM morphology characterization and particle size distribution of ordered mesoporous silica microspheres with **(a, d)** 3 μm, **(b, e)** 4 μm, and **(c, f)** 5 μm particle size. The scale bar is 10 μm.

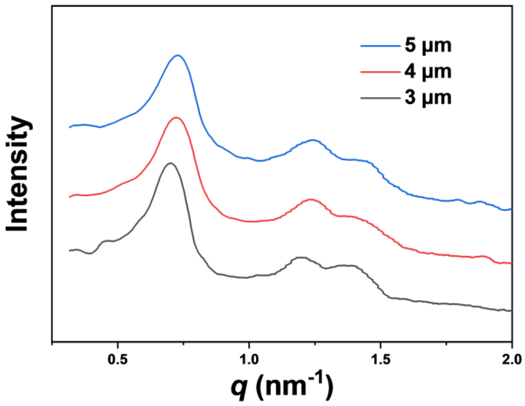

Supplementary Figure 14. SAXS patterns of ordered mesoporous silica microspheres with different particle sizes.

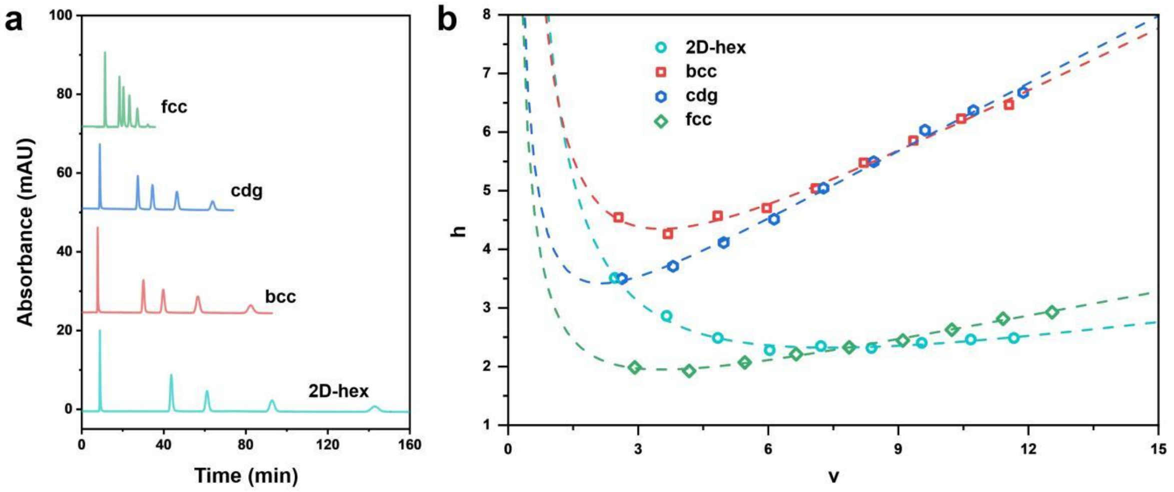

Supplementary Figure 15. Performance comparison between microspheres with different ordered mesoporous structures, i.e., 2D hexagonal (2D-hex), cubic double gyroidal (cdg), body centered cubic (bcc) and face centered cubic (fcc) structures. **a** Chromatograms of an alkylbenzene mixture on 15 cm × 100 μm i.d. capillary columns. Analytes: thiourea, methyl-, ethyl-, propyl-, and butylbenzenes (in order of elution); mobile phase: 60:40 v/v ACN/H_2O; flow rate: 100 nL/min; UV detection: 214 nm. **b** Knox curves of microspheres with different ordered mesoporous structures.

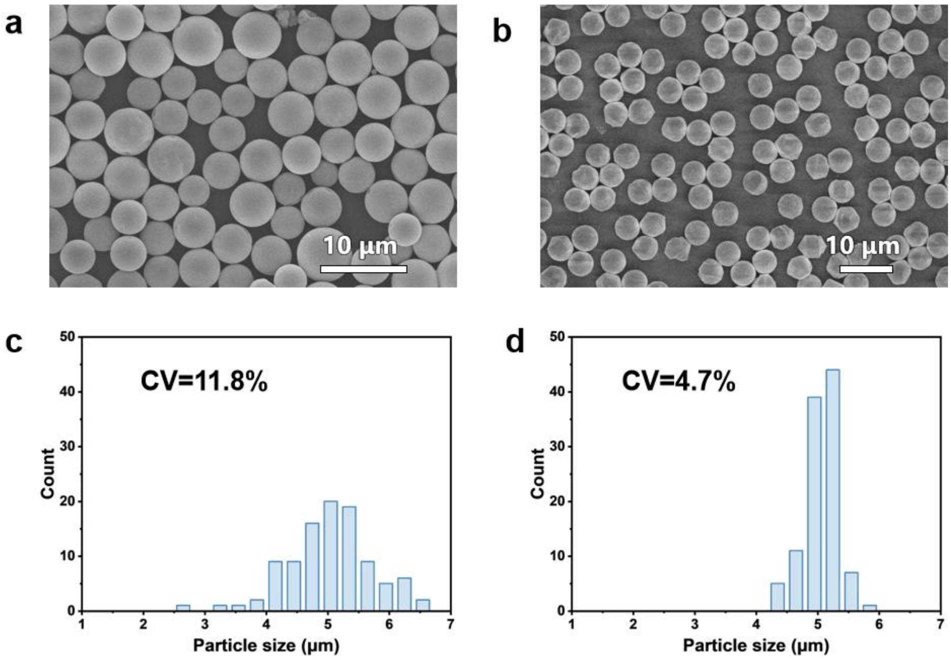

Supplementary Figure 16. Comparison of commercial totally porous particles and self-made ordered mesoporous particles. SEM morphology characterization and particle size distribution of the **(a, c)** commercial totally porous particles and **(b, d)** self-made ordered mesoporous particles.

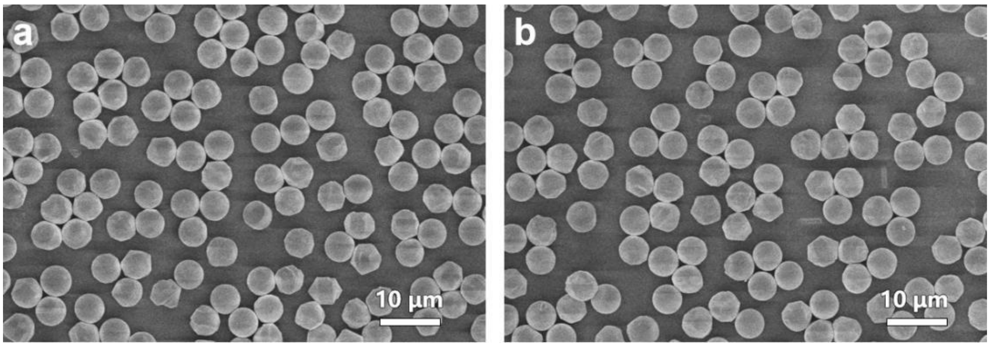

Supplementary Figure 17. Mechanical stability test of ordered mesoporous microspheres. SEM morphology characterization of ordered mesoporous silica microspheres **(a)** before and **(b)** after high pressure running test.

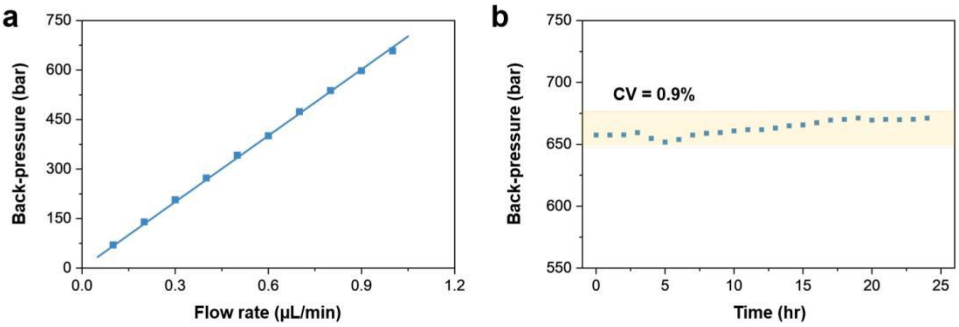

Supplementary Figure 18. Mechanical stability test of ordered mesoporous particles. a Back pressure as a function of flow rate; **b** Back pressure fluctuation during 24 hr running at the flow rate of 1.0 µL/min. Column: 27 cm × 100 µm i.d.; mobile phase: 50:50 v/v MeOH/H_2O.

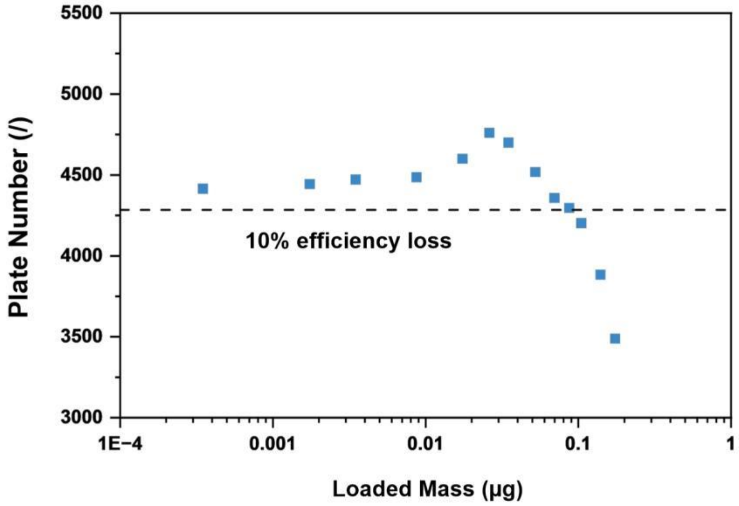

Supplementary Figure 19. Column efficiency as the function of loaded mass for methylbenzene on 15 cm × 100 μm i.d. capillary columns. Mobile phase: 60:40 v/v ACN/H_2O; flow rate: 800 nL/min; UV detection: 214 nm. The efficiency loss was maintained within ~10% for solute loading mass up to 0.087 μg.

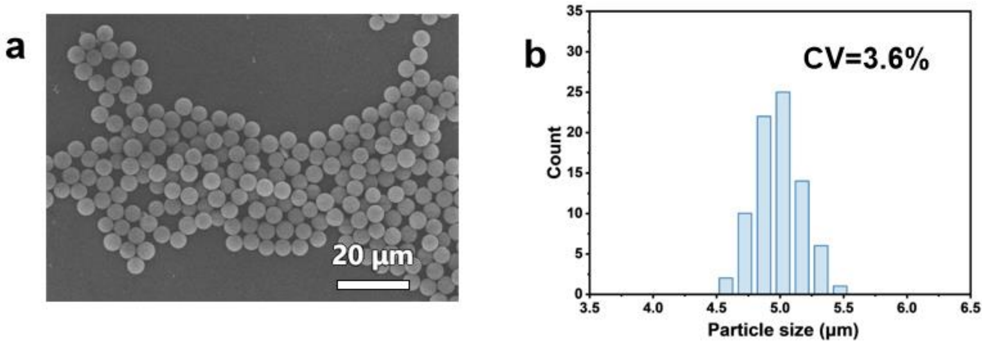

Supplementary Figure 20. Characterization of homemade totally porous silica microspheres. a SEM morphology characterization and **(b)** particle size distribution of the homemade totally porous silica microspheres.

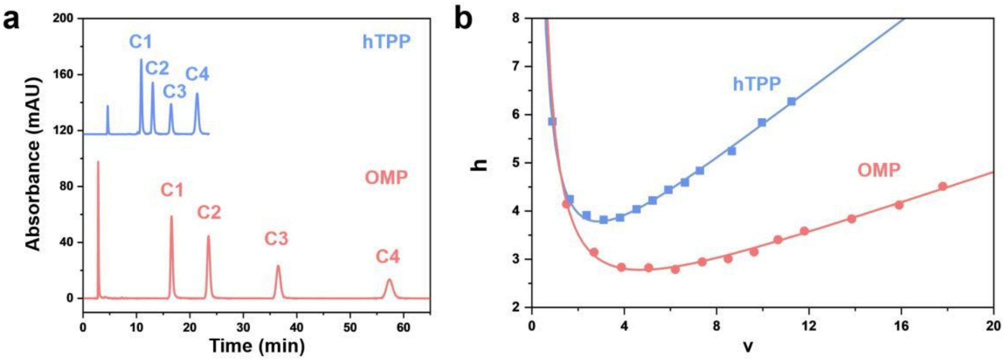

Supplementary Figure 21. Performance comparison between monodisperse ordered mesoporous particle and homemade totally porous particle. a Chromatograms of an alkylbenzene mixture on 15 cm long OMP and hTPP columns. Analytes: thiourea, methyl-, ethyl-, propyl-, and butylbenzenes (in order of elution); mobile phase: 60:40 v/v ACN/H_2O; UV detection: 214 nm. **b** Knox curves of OMP and hTPP.

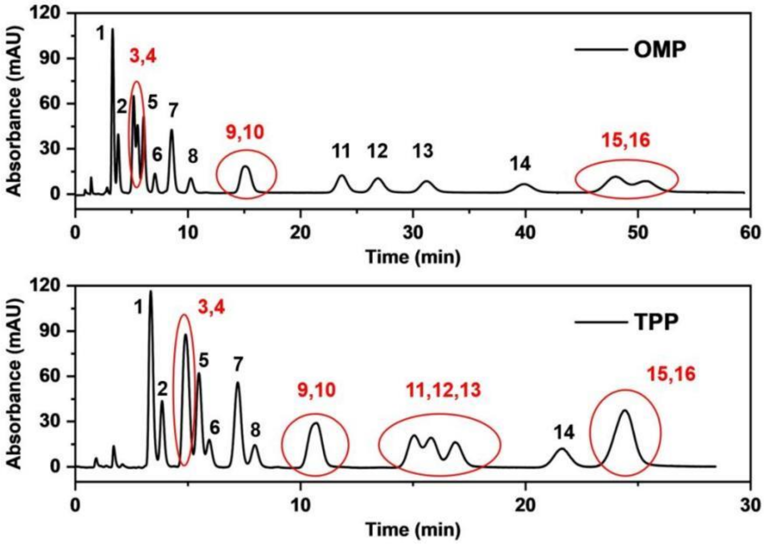

Supplementary Figure 22. Separation of a standard polycyclic aromatic hydrocarbon mixture. Peak identities: 1, naphthalene; 2, acenaphthylene; 3, fluorene; 4, acenaphthene; 5, phenanthrene; 6, anthracene; 7, fluoranthene; 8, pyrene; 9, chrysene; 10, benz(a)anthracene; 11, benzo(b)fluoranthene; 12, benzo(k)fluoranthene; 13, benzo(a)pyrene; 14, dibenz(a,h)anthracene; 15, indeno(1,2,3-cd)pyrene; and 16, benzo(g,h,i)perylene. The peaks marked in red failed to achieve effective separation.

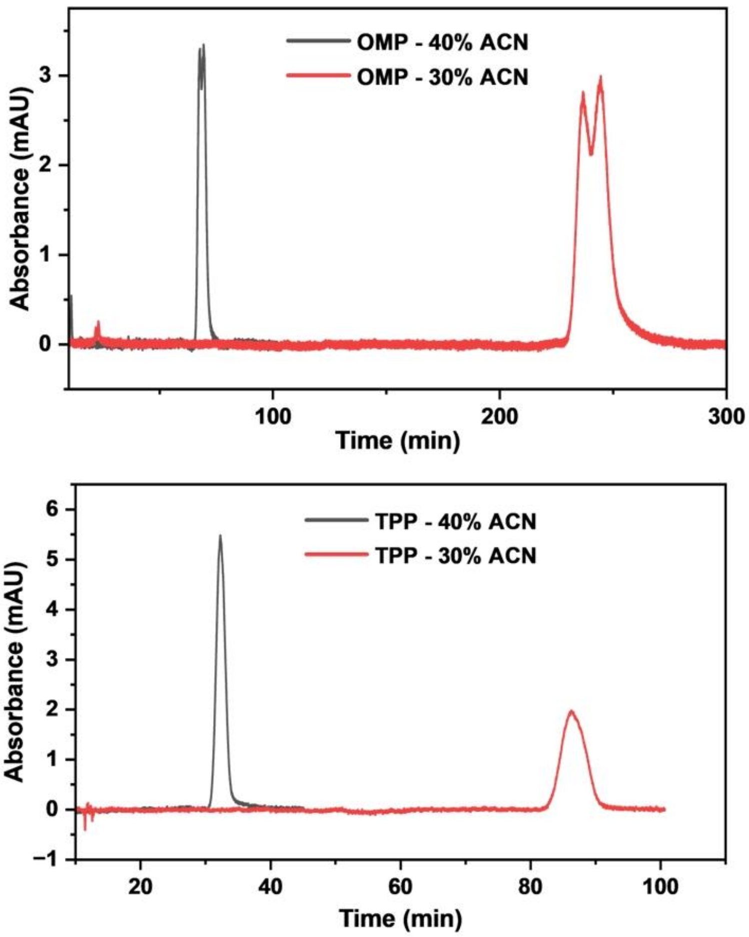

Supplementary Figure 23. Separation of 1,4-dibromobenzene and 1,4-dibromobenzene-d_4.

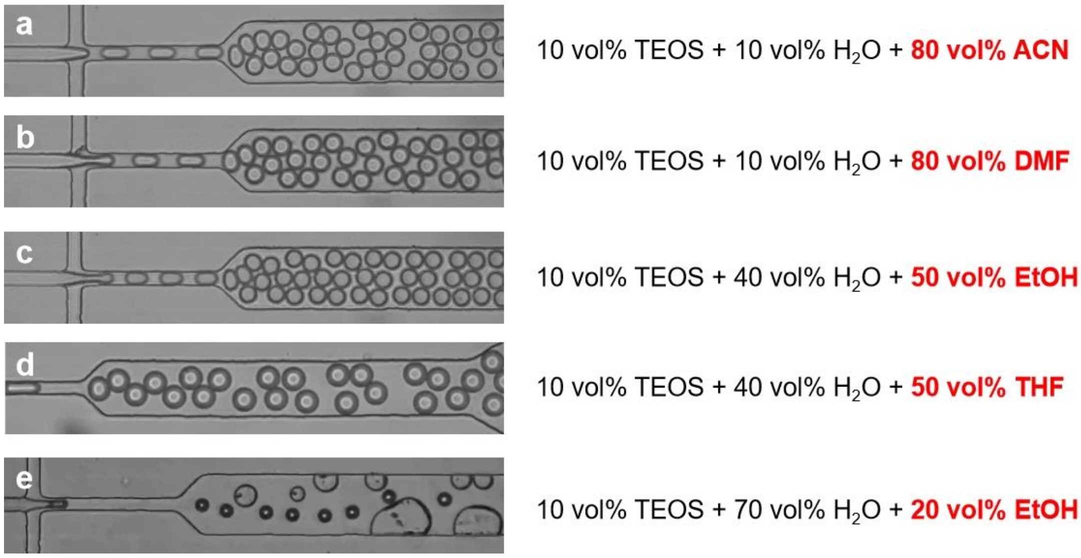

Supplementary Figure 24. Droplet generation with dispersed phases composed of various organic solvents (marked on the right). The continuous phases were fluorinated oil-HFE-7500 with **(a-d)** 1 wt% PFPE-*b*-PEG-*b*-PFPE or **(e)** n-hexadecane with 3 wt% Span80.

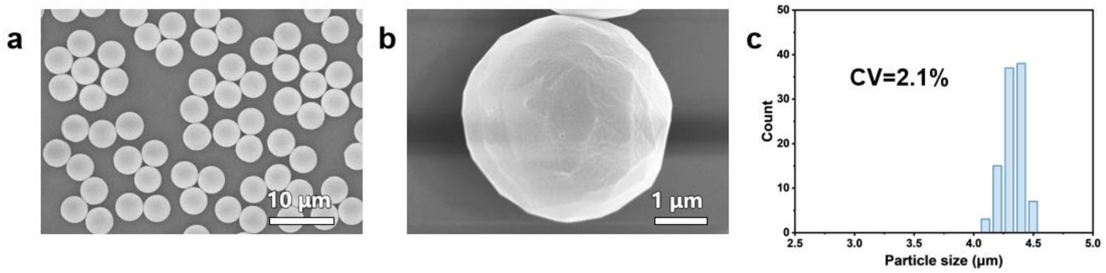

Supplementary Figure 25. Characterization of ethyl-bridged silica microspheres. a, b SEM morphology characterization and **(c)** particle size distribution of the mesoporous ethyl-bridged silica microspheres templated by P123.

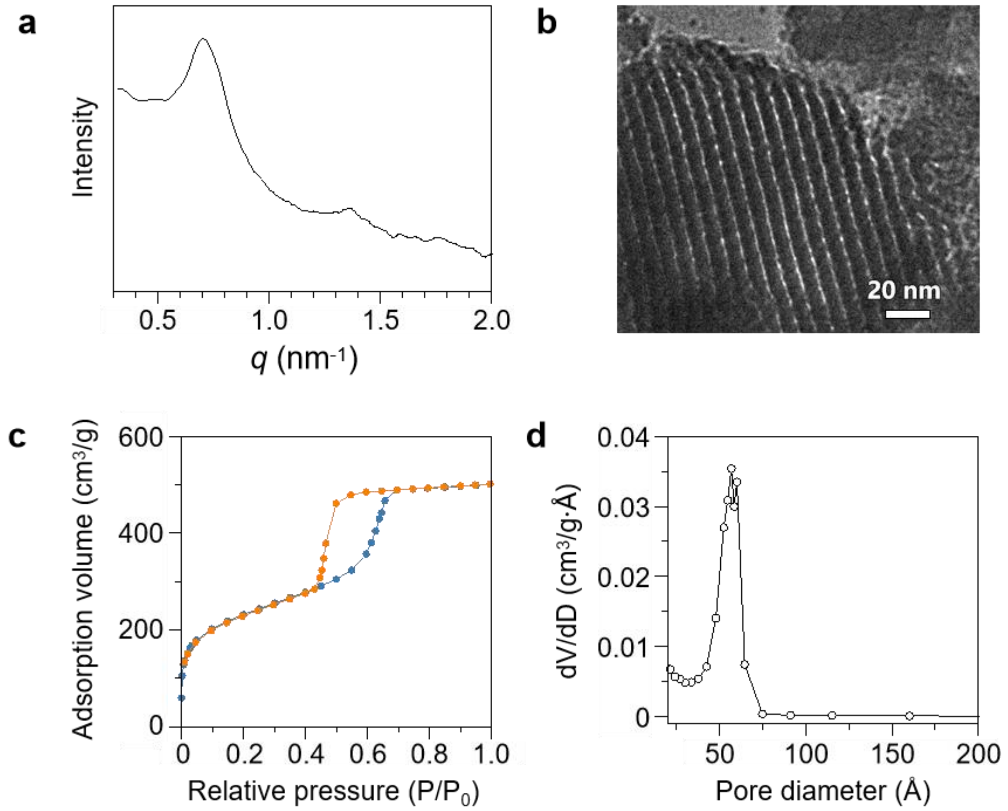

Supplementary Figure 26. Porosity characterization of the mesoporous ethyl-bridged silica microspheres templated by P123. a SAXS pattern, **(b)** TEM characterization, **(c)** nitrogen adsorption - desorption isotherms and **(d)** BJH adsorption pore size distribution.

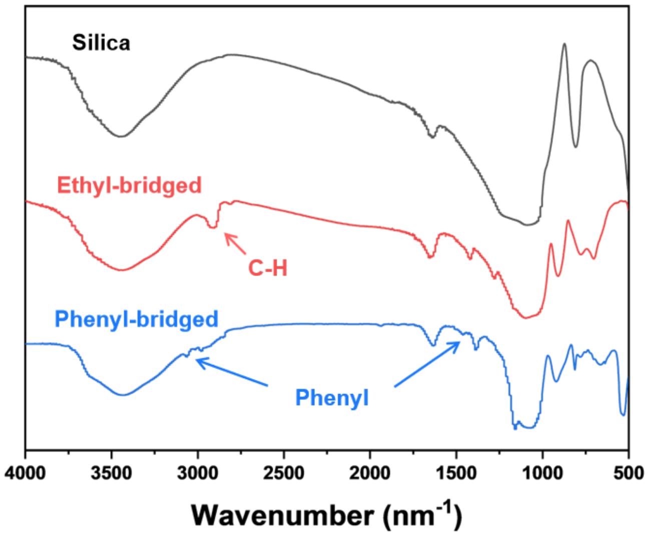

Supplementary Figure 27. FT-IR spectra of mesoporous ethyl-bridged and phenyl-bridged silica microspheres.

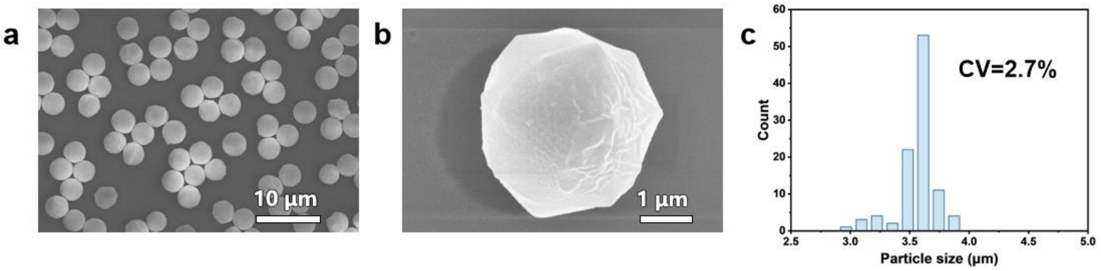

Supplementary Figure 28. Characterization of phenyl-bridged silica microspheres. a, b SEM morphology characterization and **(c)** particle size distribution of the mesoporous phenyl-bridged silica microspheres templated by P123.

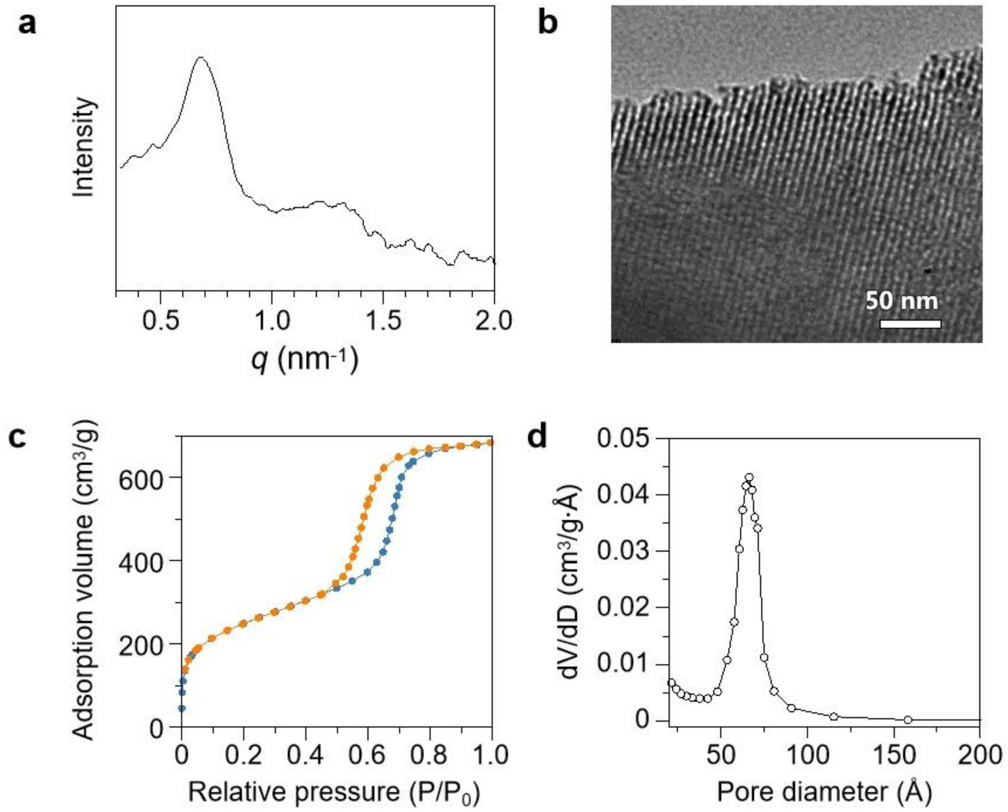

Supplementary Figure 29. Porosity characterization of the mesoporous phenyl-bridged silica microspheres templated by P123. a SAXS pattern, **(b)** TEM characterization, **(c)** nitrogen adsorption - desorption isotherms, and **(d)** BJH adsorption pore size distribution.

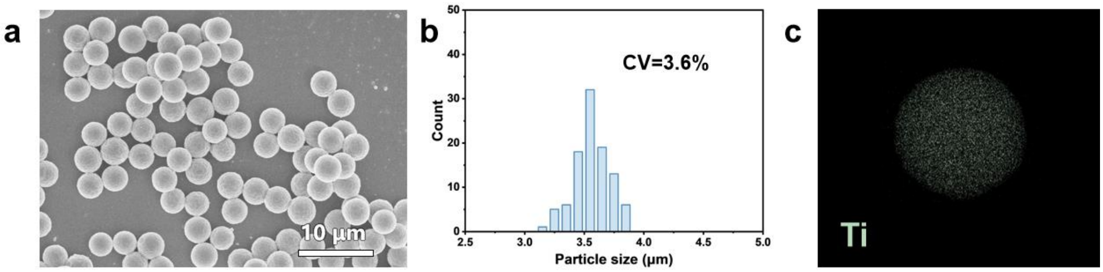

Supplementary Figure 30. Characterization of the mesoporous titanium dioxide microspheres. a SEM morphology characterization, **(b)** particle size distribution, and **(c)** EDS elemental mapping of the mesoporous titanium dioxide microspheres templated by P123.

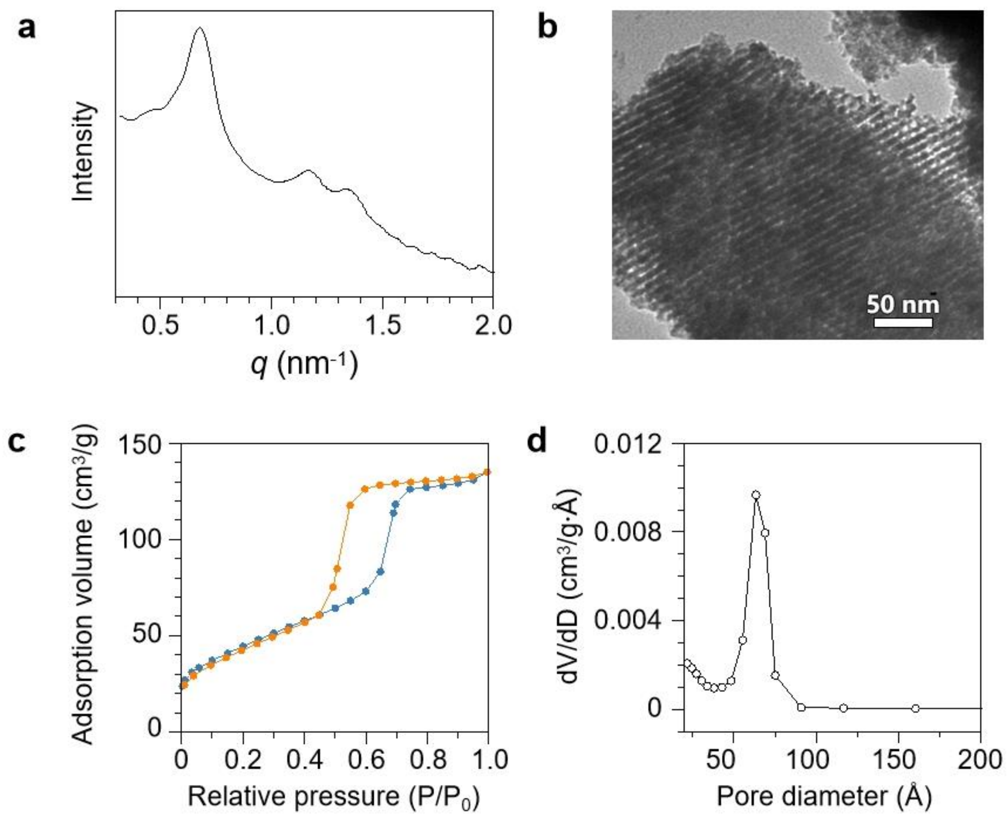

Supplementary Figure 31. Porosity characterization of the mesoporous titanium dioxide microspheres templated by P123. a SAXS pattern, **(b)** TEM characterization, **(c)** nitrogen adsorption - desorption isotherms, and **(d)** BJH adsorption pore size distribution.

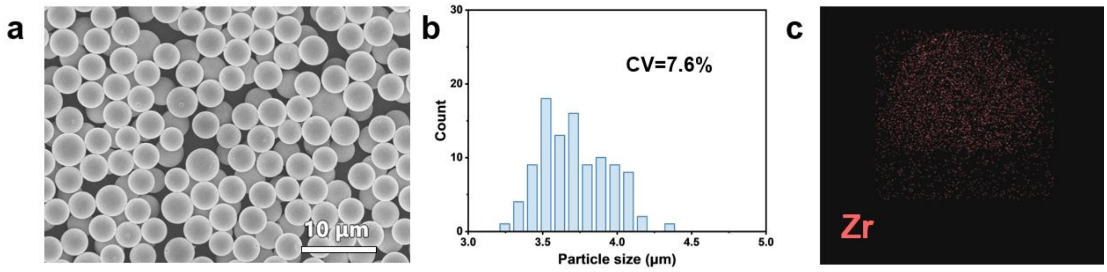

Supplementary Figure 32. Characterization of the mesoporous zirconium dioxide microspheres. **a** SEM morphology characterization, **(b)** particle size distribution, and **(c)** EDS elemental mapping of the mesoporous zirconium dioxide microspheres templated by P123.

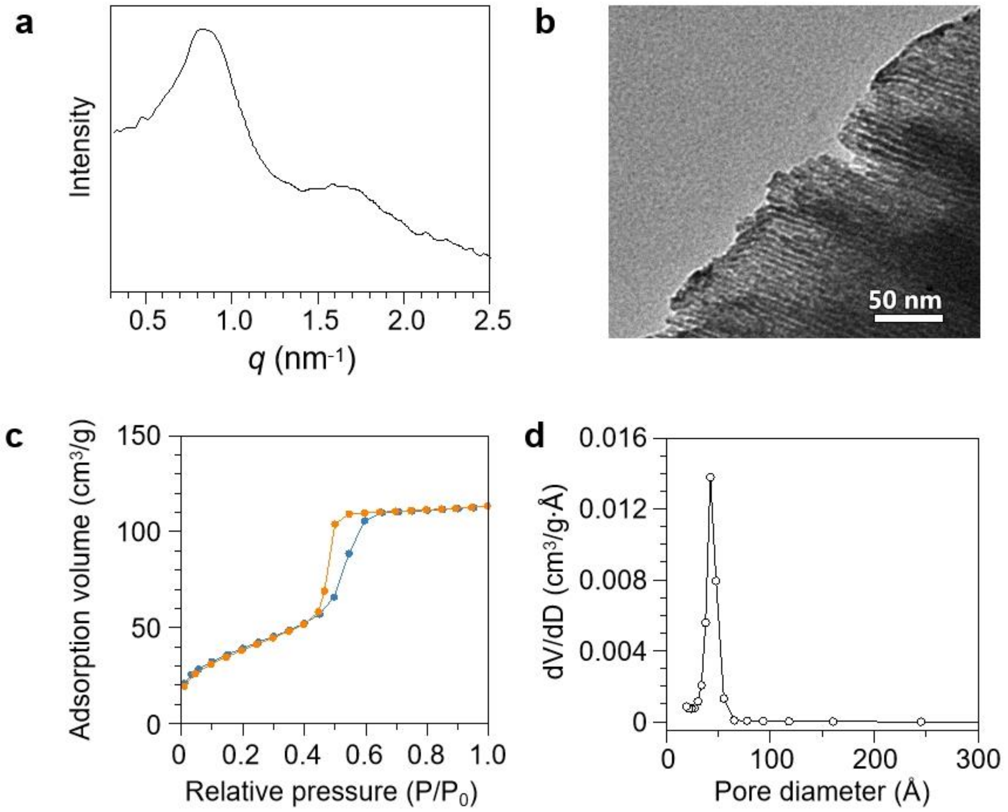

Supplementary Figure 33. Porosity characterization of the mesoporous zirconium dioxide microspheres templated by P123. a SAXS pattern, **(b)** TEM characterization, **(c)** nitrogen adsorption - desorption isotherms, and **(d)** BJH adsorption pore size distribution.

Supplementary Table 1. Porous structure properties of ordered mesoporous silica microspheres templated by P123 with different hydrothermal treatments.

	80°C ethanol extraction	100°C -24 h	120°C -24 h	130°C - 24 h	130°C - 48 h	130°C - 72 h
**Specific surface area (m^2/g)**	515	686	638	588	546	541
**Micropore specific surface area (m^2/g)**	105	161	42	0	0	0
**Mesopore specific surface area (m^2/g)**	410	525	596	588	546	541
**Pore volume (cm^3/g)**	0.42	0.74	0.91	0.95	0.93	0.97
**Micropore volume (cm^3/g)**	0.05	0.08	0.02	0	0	0
**Mesopore volume (cm^3/g)**	0.37	0.66	0.89	0.95	0.93	0.97
**Pore size (nm)**	4.4	6.9	7.1	7.7	8.0	8.4
**Cell parameter (nm)**	8.72	9.94	9.80	10.08	10.18	10.26
**Wall thickness (nm)**	4.32	3.04	2.70	2.38	2.18	1.86

Supplementary Table 2. Porous structure properties of ordered mesoporous silica microspheres templated by PEO_{125}-b-$PMMA_{249}$ with different hydrothermal treatments.

	Direct calcination	100°C -24h	130°C -24h	130°C -72h
**Specific surface area (m^2/g)**	450	463	448	215
**Micropore specific surface area (m^2/g)**	201	93	44	20
**Mesopore specific surface area (m^2/g)**	249	370	404	205
**Pore volume (cm^3/g)**	0.46	0.66	0.90	0.67
**Micropore volume (cm^3/g)**	0.10	0.04	0.02	0.01
**Mesopore volume (cm^3/g)**	0.36	0.62	0.88	0.66
**Pore size (nm)**	15.0/6.1	18.5/8.8	20.9/11.2	22.6/13.8
**Cell parameter (nm)**	43.26	46.41	47.14	47.73
**Wall thickness (nm)**	15.59	14.32	12.43	11.15

Supplementary Table 3. Porous structure properties of ordered mesoporous silica microspheres templated by different P123 amounts.

**P123/TEOS mass ratio**	0.210	0.253	0.295	0.337	0.379	0.421	0.463	0.505
**Specific surface area (m^2/g)**	651	664	681	723	603	649	630	440
**Micropore specific surface area (m^2/g)**	73	98	105	120	35	43	36	20
**Mesopore specific surface area (m^2/g)**	578	566	576	603	568	606	594	420
**Pore volume (cm^3/g)**	0.33	0.51	0.70	0.90	1.04	1.05	1.09	0.88
**Micropore volume (cm^3/g)**	0.03	0.05	0.05	0.05	0.01	0.02	0.01	0.01
**Mesopore volume (cm^3/g)**	0.30	0.46	0.65	0.85	1.03	1.03	1.08	0.87
**Pore size (nm)**	3.0	5.4	6.1	7.1	8.5	8.5	9.2	8.6
**Cell parameter (nm)**	NA	NA	9.9	10.5	10.5	10.5	10.5	10.5
**Wall thickness (nm)**	NA	NA	3.8	3.4	2	2	1.3	1.9

Supplementary Table 4. Porous structure properties of ordered mesoporous silica microspheres templated by different PEO_{125}-*b*-$PMMA_{249}$ amounts.

PEO-PMMA/TEOS	10 wt%	20 wt%	30 wt%	40 wt%
**Specific surface area (m^2/g)**	375	448	381	420
**Micropore specific surface area (m^2/g)**	29	44	53	11
**Mesopore specific surface area (m^2/g)**	346	404	328	409
**Pore volume (cm^3/g)**	0.63	0.90	1.27	1.64
**Micropore volume (cm^3/g)**	0.01	0.02	0.02	0
**Mesopore volume (cm^3/g)**	0.62	0.88	1.25	1.64
**Pore size (nm)**	20.3	20.9	21.9	22.8
**Cell parameter (nm)**	50.77	47.14	47.14	47.14
**Wall thickness (nm)**	15.60	12.43	11.43	10.53

Supplementary Table 5. Porous structure properties of ordered mesoporous silica microspheres templated by P123 with different particle sizes.

**Particle sizes (µm)**	3.0	4.1	5.0
**Specific surface area (m^2/g)**	637	577	519
**Pore volume (cm^3/g)**	0.89	0.95	0.89
**Pore size (nm)**	7.5	7.7	7.6
**Cell parameter (nm)**	10.21	10.08	9.94
**Wall thickness (nm)**	2.71	2.38	2.34

Supplementary Table 6. Properties of C18-bonding packing materials.

**Packing material**	OMP	TPP
**Specific surface area (m^2/g)**	166	215
**Pore size (nm)**	4.1	11.5
**Pore volume (cm^3/g)**	0.26	0.63
**Carbon load (%)**	22.5	14.8
**Particle size (µm)**	5	5
**Stationary phase**	C18	C18
**Ligand density (µmol/m^2)**	2.65	< 2.30

Supplementary Table 7. Comparation of Knox coefficients and reduced plate heights obtained with TiTAN-synthesized ordered mesoporous particles, commercial totally porous particles, and homemade totally porous particles.

	h_{min}	a	b	c
OMP	2.78	0.65	4.83	0.14
TPP	4.08	1.05	5.14	0.32
hTPP	3.82	1.02	4.09	0.32

Supplementary Table 8. Properties of homemade totally porous particle.

**Material**	Silica	C18-Silica
**Specific surface area (m^2/g)**	347	208
**Pore size (nm)**	8.2	7.3
**Pore volume (cm^3/g)**	0.78	0.55
**Carbon load (%)**	0	10.3
**Particle size (μm)**	5	5
**Coefficient variance of particle size (%)**	3.6	3.6
**Ligand density (μmol/m^2)**	0	1.58

Supplementary Movie

Supplementary Movie 1. Solvent evaporation of droplets.

Supplementary Movie 2. Droplet generation by using dispersed and continuous phases with various compositions.

References

1. Wei, J. et al. Solvent evaporation induced aggregating assembly approach to three-dimensional ordered mesoporous silica with ultralarge accessible mesopores. *J. Am. Chem. Soc.* **133**, 20369-20377 (2011).

2. Holtze, C. et al. Biocompatible surfactants for water-in-fluorocarbon emulsions. *Lab. Chip* **8**, 1632-1639 (2008).

3. Matochko, W. L. et al. Uniform amplification of phage display libraries in monodisperse emulsions. *Methods* **58**, 18-27 (2012).

4. Sun, K. et al. Microfluidic precision manufacture of high performance liquid chromatographic microspheres. *Angew. Chem. Int. Ed.* **64**, e202418642 (2025).
