# Supplementary material for: Template-in-template assembly nanostructured microspheres for high performance chromatography
Source: Nat Commun. 2026 Feb 4;17:430. doi: 10.1038/s41467-026-68362-y (PMC12873412; doi:10.1038/s41467-026-68362-y)
Supplement: Supplementary file 2 — Description of Additional Supplementary Files [file 41467_2026_68362_MOESM2_ESM.pdf]

Description of Additional Supplementary Files

Supplementary Movie 1: Solvent evaporation of droplets.

Supplementary Movie 2: Droplet generation by using dispersed and continuous phases with various compositions.
